# Prevalence of malaria and hepatitis B among pregnant women in Northern Ghana: Comparing RDTs with PCR

**DOI:** 10.1371/journal.pone.0210365

**Published:** 2019-02-06

**Authors:** Nsoh Godwin Anabire, Paul Armah Aryee, Abass Abdul-Karim, Issah Bakari Abdulai, Osbourne Quaye, Gordon Akanzuwine Awandare, Gideon Kofi Helegbe

**Affiliations:** 1 West African Centre for Cell Biology of Infectious Pathogens (WACCBIP), University of Ghana, Legon- Accra, Ghana; 2 Department of Biochemistry, Cell and Molecular Biology, University of Ghana, Legon, Accra-Ghana; 3 Department of Biochemistry & Molecular Medicine, School of Medicine and Health Sciences, University for Development Studies, Tamale-Ghana; 4 Department of Nutritional Sciences, School of Allied Health Sciences, University for Development Studies, Tamale-Ghana; 5 Zonal Public Health Laboratory, Tamale Teaching Hospital, Tamale-Ghana; 6 Tamale West Hospital Laboratory; Clear Lens Diagnostic Laboratory, Tamale-Ghana; Centre de Recherche en Cancerologie de Lyon, FRANCE

## Abstract

**Background:**

High prevalence of malaria and hepatitis B has been reported among pregnant women in Ghana. In endemic areas, the diagnoses of malaria and hepatitis B among pregnant women on antenatal visits are done using histidine-rich protein 2 (HRP2) and hepatitis B surface antigen (HBsAg) rapid diagnostic tests (RDTs), respectively, which are, however, reported to give some false positive results. Also, socio-economic determinants have been drawn from these RDTs results which may have questionable implications. Thus, this study was aimed at evaluating the prevalence of malaria and hepatitis B by comparing RDTs with polymerase chain reaction (PCR) outcomes, and relating the PCR prevalence with socio-economic status among pregnant women in Northern Ghana.

**Methods:**

We screened 2071 pregnant women on their first antenatal visit for *Plasmodium falciparum* and hepatitis B virus (HBV) using HRP2 and HBsAg RDTs, and confirming the infections with PCR. Socio-economic and obstetric information were collected using a pre-tested questionnaire, and associations with the infections were determined using Pearson’s chi-square and multinomial logistic regression analyses at a significance level of *p*<0.05.

**Results:**

The prevalence of the infections by RDTs/PCR was: 14.1%/13.4% for *P*. *falciparum* mono-infection, 7.9%/7.5% for HBV mono-infection, and 1.9%/1.7% for *P*. *falciparum*/HBV co-infection. No statistical difference in prevalence rates were observed between the RDTs and PCRs (χ^2^  =  0.119, *p* = 0.73 for malaria and χ^2^  =  0.139, *p* = 0.709 for hepatitis B). Compared with PCRs, the sensitivity/specificity of the RDTs was 97.5%/99.1% and 97.9%/99.4% for HRP2 and HBsAg respectively. Socio-economic status was observed not to influence HBV mono-infection among the pregnant women (educational status: AOR = 0.78, 95% CI = 0.52–1.16, *p* = 0.222; economic status: AOR = 1.07, 95% CI = 0.72–1.56, *p* = 0.739; financial status: AOR = 0.66, 95% CI = 0.44–1.00, *p* = 0.052). However, pregnant women with formal education were at a lower risk for *P*. *falciparum* mono-infection (AOR = 0.48, 95% CI  =  0.32–0.71, *p*<0.001) and *P*. *falciparum*/HBV co-infection (AOR = 0.27, 95% CI  =  0.11–0.67, *p* = 0.005). Also those with good financial status were also at a lower risk for *P*. *falciparum* mono-infection (AOR = 0.52, 95% CI  =  0.36–0.74, *p*<0.001).

**Conclusion:**

Our data has shown that, the RDTs are comparable to PCR and can give a representative picture of the prevalence of malaria and hepatitis B in endemic countries. Also, our results support the facts that improving socio-economic status is paramount in eliminating malaria in endemic settings. However, socio-economic status did not influence the prevalence of HBV mono-infection among pregnant women in Northern Ghana.

## Background

Malaria and hepatitis B virus (HBV) infections continue to be a major public health threats world-wide and among pregnant women. In 2016, global reports of new malaria-cases and malaria-related deaths were 216 million and 445000, respectively [1]. In Ghana, although effective interventions including Intermittent Preventive Treatment during Pregnancy (IPTp), routine malaria diagnosis of pregnant women on antenatal visit and vector management strategies have been put in place to protect pregnant women, malaria still accounts for 17.6% of Out Patient Department (OPD) attendance, 13.7% of admissions and 3.4% of maternal deaths [2]. On its part, HBV infection causes significant morbidity and mortalityaffecting2 billion people and killing over half a million across the world [3–5]. The prevalence of HBV infections in Ghana is 12.3%, and in pregnant women, a national prevalence of 13.1% is recorded [6]. To reduce this burden, hepatitis B vaccination, which is safe for administration in pregnancy, has remained the key prevention strategy for protecting pregnant women against the disease.

The reported prevalence rates of malaria and hepatitis B in Ghana are based on the use of histidine-rich protein 2 (HRP2) and hepatitis B surface antigen (HBsAg) rapid diagnostic tests (RDTs), respectively, which are thought to have sensitivity and specificity drawbacks. In a hospital based study where PCR was used as a gold standard, the sensitivity/specificity of HRP2 RDTs was 56.4/90.0% [7]. On the hand, most studies have evaluated the diagnostic accuracy of HBsAg RDTs using laboratory-based enzyme immunoassays (EIAs) as the reference standard, and the pooled sensitivity/specificity reported are 90.0%/99.5% [8]. Though diagnostics escape is widely ascribed to reduced sensitivity, reduced specificity accounts for false positive test results and over-diagnosis of diseases. In particular, false positive HRP2 results have been reported in endemic regions [9], and reasons have been ascribed to prolonged presence of HRP2 after parasite clearance[10, 11] or cross reactivity with other infections such as typhoid fever, hepatitis C and schistosomiasis [12–14]. Also, concerns about false positive HBsAg reactivity has been reported, which may be associated with recent hepatitis B vaccination [15, 16]. However, it is not known whether false positive HRP2 and HBsAg results could be a contributing factor to the high prevalence rates of the diseases reported amongst pregnant women in Ghana. The study therefore compared the RDTs prevalence data with that of polymerase chain reaction (PCR), a more sensitive and specific diagnostic tool [7].

Factors including gravidity, young maternal age, use of treated bed nets, and use of antimalarial chemotherapy and prophylaxis have been implicated to influence the prevalence of malaria among pregnant women [17, 18]. On the part of HBV infection, risky sexual behaviors are observed to be key predisposing factors [19, 20].These aforementioned factors may either be directly or indirectly influenced by one’s socio-economic status (SES); defined by factors such as education, occupation, income, and value of individual’s dwelling place. In particular, low SES is associated with higher HBV prevalence in both developed and developing countries [21, 22]. Improving the SES of people in malaria endemic areas has been shown to be a paramount factor in eliminating malaria [23]. In many studies, the evaluation of the influence of SES on the prevalence of malaria or hepatitis B are drawn from RDT results. Therefore, this study aimed at associating PCR determined prevalence of malaria and hepatitis B, with SES among pregnant women in northern Ghana.

## Methods

### Study design and sampling sites

**Fig 1:**
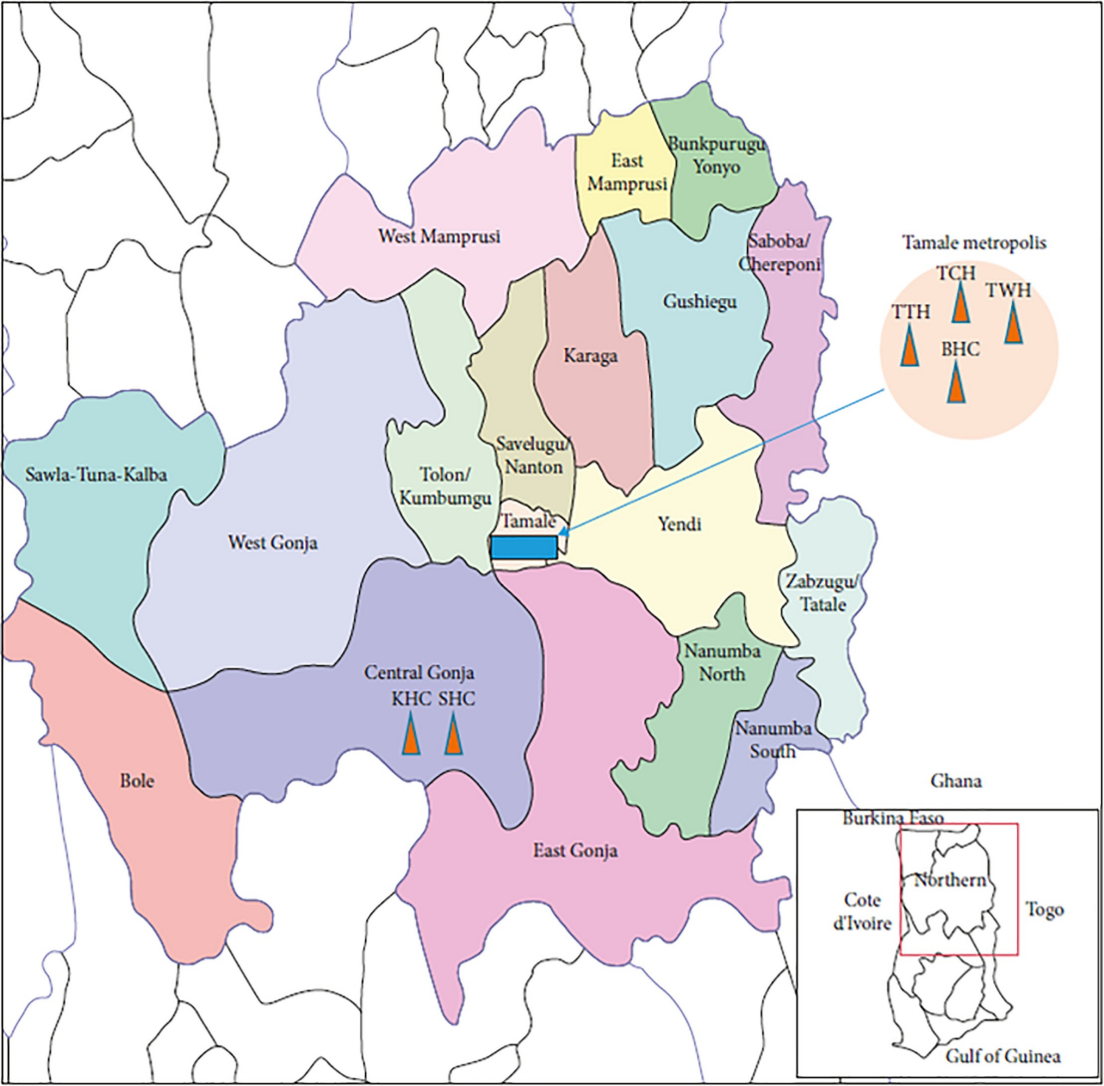
Map of Ghana showing the locations of the study sites (triangles in orange). The study sites are located in the Northern Region of Ghana. TTH: Tamale Teaching Hospital, TCH: Tamale Central Hospital, TWH: Tamale West Hospital, and BHC: Bilpella Health Centre are found in the Tamale Metropolis. While SHC: Sankpala Health Centre and KHC: Kosawgu Health Centre are found in the Central Gonja District (S1 File).

This was a cross-sectional study conducted from October 2016 to February 2017among pregnant women on their first antenatal care (ANC) visit at six different health facilities (three hospitals and three health centres) in the Northern Region of Ghana (Fig 1).The pregnant women were recruited at random, depending on the time (i.e. whether during first, second or third trimester of pregnancy) they booked for ANC. The Northern Region was particularly chosen because, the Region recorded over 100% of ANC performance from 2012–2015 compared to the national performance of about 87% [24].Out of all the districts (Fig 1), Tamale Metropolis and Central Gonja were selected at random and used for the study.

### Ethical consideration

Informed consent was obtained from each study participant after an explanation of the purpose, benefits, and risks of the study was provided. The ethics committee of the Tamale Teaching Hospital approved the study (Approval ID: TTHERC/21/04/16/02).

### Determination of sample size

The sample size was calculated based on the national prevalence of malaria [2] and that of hepatitis B [6] in pregnancy. The Cochran's formula, $\frac{z^{2}pq}{E^{2}}$ was used, where z = 95% confidence interval, p = odds with the disease, q = odds without the disease and E = margin of error.

Sample size (n) = Screening size based on malaria prevalence (n_malaria_)+ Screening size based on hepatitis B prevalence (n_HBV_)

n_malaria_ = $\frac{z^{2}pq}{E^{2}}$, z = 1.96, p = 0.176 (17.6%), q = 0.824 (82.4%), and E = 0.015 (1.5%).

n_HBV_ = $\frac{z^{2}pq}{E^{2}}$, z = 1.96, p = 0.131 (13.1%), q = 0.869 (86.9%), and E = 0.015 (1.5%).

n = [(1.96^2^ x 0.176 x 0.824)/0.015^2^] + [(1.96^2^ x 0.131 x 0.869)/0.015^2^] = 2258

The number of pregnant women recruited for the study was 2258. However, due to missing data of 187 pregnant women, 2071 pregnant women were used in the analysis.

### Collection of socio-economic, obstetric and demographic data

Socio-economic and obstetric information of each study participant was obtained using a structured questionnaire. Training was given to six (6) qualified enumerators on the questionnaire, which was then pre-tested. The Questionnaire enumerators were research assistants at the University for Development Studies, and Health personnel at the Zonal Public Health Laboratory, Tamale Teaching Hospital, Ghana. The interviews were administered face-to-face with each pregnant woman. The socio-economic data collected included marital, educational, economic and financial statuses. Educational status was assessed as having any form of formal education (basic, secondary or tertiary education) or not. Economic status was assessed from the occupation of the participants as being involved in economic activity (farmer, trader, teacher, hairdresser, seamstress) or not (house wife, student). Financial status was assessed based on the minimum daily wage of GH¢ 8.00 as stipulated by the Government of Ghana in January 2016 (http://www.ghana.gov.gh/index.php/media-center/news/2023-%09government-announces-increment-in-minimum-wage-salaries-for-public-workers), and was assigned as good if they earned ≥GH¢ 8.00/day, or poor if they earned <GH¢ 8.00/day. Obstetric history which included gestational age (first, second or third trimesters) and gravidity (primigravidae or multigravidae), and participants’ age were obtained from patients’ antenatal records books.

### Clinical samples and diagnoses

Trained medical laboratory scientists were recruited at each screening centre to aid in the collection of venous blood samples from the study participants. Approximately 2 mL of venous blood was collected from each participant into an EDTA tube for testing and analyses. A volume of 75 μL was spotted on a Whatmann filter paper, dried, and sealed in clean zip-lock plastic bag with desiccants. The dry blood spots (DBS) were stored under room temperature. A week after the sampling period, the DBS were transported by the investigator (Nsoh Godwin Anabire) to the Infectious Disease Laboratory (IDRL) at the West African Centre for Cell Biology of Infectious Pathogens (WACCBIP), University of Ghana, for molecular detection of plasmodium species and HBV. At the IDRL, the DBS were stored at room temperature for a week prior to the molecular detections of the pathogens by the investigator. The RDT cassette, CareStart HRP-2 (Access BioInc., New Jersey, USA) was used in diagnosing malaria. Commercially available hepatitis B surface antigen (HbsAg) test strips (Intec Products Inc., Xiamen, China) were used for serological testing for HBV. For both tests, the testing, results reading and interpretations were done with strict adherence to the manufacturer’s protocol. As a quality assurance procedure, two RDTs from each lot containing 50 strips and 25 cassettes were confirmed by testing with known negative and positive samples before such lots were used in the testing process.

### Extraction of DNA

The chelex extraction method was used to extract DNA from the DBS. Briefly, DBS containing 75 μL of blood were excised into 1.5 mL eppendorf tubes and soaked overnight with 1 mL of 0.5% saponin in 1X phosphate buffered saline (PBS) solution. The eppendorf tubes with the contents were centrifuged at 14000 rpm for 2 mins, saponin suctioned, and the contents of the eppendorf tubes were washed with 1 mLof 1X PBS and suctioned. The washing step was repeated until no haem or red colour was seen on the filter papers. A volume of 50 μL of 20% chelex and 100 μL nuclease free water were added to the tubes, and incubated in a boiling water bath for 20 mins. The tubes were centrifuged at 14000 rpm for 2 mins and 100 μL of the resulting supernatant, which contained DNA, was pipetted into a 96 well plate and stored at -20°C until used.

### Polymerase chain reaction (PCR) for detection of plasmodium parasites and HBV

Plasmodium species were detected using a nested PCR method previously described [25, 26]. The first round PCR reaction used forward and reverse primers that amplified the small sub-unit ribosomal genes of plasmodium parasites, while the second round PCR used species-specific forward and reverse primers to detect *P*. *falciparum*,*P*. *vivax*, *P*. *malariae*, and *P*. *ovale*. For both rounds of PCR, a total reaction volume of 20 μL was used which contained 10X PCR buffer, 25 mM MgCl_2,_10 μM primers, 10 μM deoxynucleotides, 1 U Taq DNA polymerase (Qiagen, Hilden- Germany), nuclease free water and DNA sample. Cycling conditions of initial denaturation step of 95°C for 5 minutes, followed by 35 cycles of denaturation at 94°C for 30 Sec, annealing at 58°C for 1 minute, elongation at 68°C for 1.5 minutes, and final elongation at 68°C for 5 minutes were used in both assays. For the detection of HBV, forward and reverse primers for a previously described PCR assay that amplifies the S-gene of HBV genome were used (27). A total reaction volume of 10 μL consisting of10μMprimers, PerfeCTa syber green SuperMix, Low ROX (Quanta biosciences), nuclease free water and DNA sample, was used. The cycling conditions were: denaturation at 95°C for 15 minutes, followed by 40 cycles that included: denaturation at 94°C for 15 seconds, annealing at 55°C for 30 seconds and elongation at 68°C for 30 seconds. For both plasmodium species and HBV detections, the PCR products were visualized in 2% agarose gels stained with 1.5 μL of ethidium bromide.

### Statistical analysis

Data were entered into Microsoft Excel 2016 (Microsoft Corporation, Redmond, USA), and proofread to ensure there were no errors. The entered data were then exported into SPSS Version 20 (IBM Corporation, Chicago, USA) and Graphpad Prism 6 (GraphPad Software Inc., San Diego, USA) for statistical analyses. All comparisons with p-value < 0.05 (two-tailed) were considered statistically significant. Age, a continuous variable, was normally distributed, and therefore was described as mean with standard deviation, and compared across the four groups of pregnant women (un-infected, *P*. *falciparum* mono-infection, HBV mono-infection, *P*. *falciparum*/HBV co-infection)by one-way analysis of variance (ANOVA). Categorical variables (gestation, gravidity and socio-economic factors) were presented as frequency and percentages, and compared by Pearson’s chi-square test. Association between socio-economic factors(education, economic activities, and financial statuses) and infection status (with either *P*. *falciparum* mono-infection, HBV mono-infection or their co-infection) was analyzed by the multinomial regression analysis, with the un-infected as the reference group. Infection status was the dependent variable while age, gestation, gravidity and the SES were the predictor variables.

## Results

### Outcome of interviews

One thousand, nine hundred and eighty-six (1986) pregnant women responded to the questionnaires (95.9% response rate). The remaining 85 pregnant refused to respond to questionnaire due to personal (n = 21, 1.0% refusal rate), religious (n = 15, 0.7% refusal rate) or no given reasons (n = 49, 2.4% refusal rate).

### Background characteristics of study participants

The average age of the pregnant women (*n*  = 2071) was 27.8±5.8 years old. Majority were married (*n*  = 1838, 92.6%), while the unmarried were either single (n = 188, 9.5%) or divorced (n = 38, 0.9%).With regards to gravidity, 78.1% (*n*  = 1617) were multigravidae, while 21.9% (*n*  = 454) were primigravidae. As at the time of antenatal visit, 62.8% (*n*  = 1300) were in the second trimester while 26.7% (*n*  = 552) and 10.6% (*n*  = 219) were in their first and third trimesters, respectively. SES was evaluated from the respondents’ educational, economic and financial statuses. Most respondents (*n*  = 1228, 61.8%) had no formal education, that notwithstanding, 62.1% (*n*  = 1234) were involved in economic activities. Of those, 44.3% (*n*  = 547) were farmers, 31.6% (*n*  = 390) were traders, 10.4% (*n*  = 128) were hairdresser, 8.7% (107) were seamstress and 5.0% (*n*  = 62) were teachers. Respondents who were not involved in any economic activities were either house wives (n = 600, 79.8%) or students (n = 152, 20.2%). Also, more than half of the respondents (*n*  = 1117, 56.2%) were in good financial standing.

### Prevalence of plasmodium parasites mono-infection, HBV mono-infection or their co-infection

A total of 2071 blood samples were tested for malaria and hepatitis B using HRP2 and HBsAg RDTs, and the infections were confirmed by PCR. The prevalence of the infections by RDTs were: 13.7% for *P*. *falciparum* mono-infection (n = 283), 7.7% for HBV mono-infection (n = 159), 1.9% for *P*. *falciparum*/HBV co-infection (n = 39). However, using PCR, the prevalence rates were: 13.4% for *P*. *falciparum* mono-infection (n = 278), 7.5% for HBV mono-infection (n = 155) and 1.7% for *P*. *falciparum*/HBV co-infection (n = 36). No statistical difference in prevalence rates were observed between the RDTs and PCRs (χ^2^  =  0.119, *p* = 0.73 for malaria and χ^2^  =  0.139, *p* = 0.709 for hepatitis B; Table 1).

**Table 1:** Comparison of RDT and PCR for malaria and hepatitis B prevalence in study participants.

Participants test results	HRP RDT n (%)	Malaria PCR n (%)	(*X*^*2*^),^α^*p*
Positive	322 (15.5%)	314 (15.2%)	*X*^*2*^ = 0.119,^α^0.73
Negative	1749 (85.5%)	1757 (84.8%)	
	HBsAg RDT n (%)	HBV PCR n (%)	
Positive	198 (9.6%)	191 (9.2%)	*X*^*2*^ = 0.139, ^α^0.709
Negative	1873 (90.4%)	1880 (90.8%)	
Malaria status by RDT	Malaria status by PCR Positive **(sensitivity)**	Negative **(specificity)**	
Positive	306 **(97.5%)**	16	
Negative	8	1741**(99.1%)**	
Hepatitis B status by RDT	Hepatitis B status by PCR Positive **(sensitivity)**	Negative **(specificity)**	
Positive	187 **(97.9%)**	11	
Negative	4	1869 **(99.4%)**	

^**α**^analyzed using Pearson’s chi-square test.

χ^2^  = Pearson’s chi-square value

*p* significant at <0.05 (2-tailed).

Sensitivity and specificity: analyzed using the chi-square trend of sensitivity and specificity on Graphpad Prism 6.

### Diagnostic performance of the RDTs

Compared with PCRs, the sensitivity/specificity were respectively 97.5%/99.1% and 97.9%/99.4% for the HRP2 and HBsAg RDTs (Table 1). The area under the receiver operator curve (ROC) was 0.983 (95% CI:0.972–0.993) for the HRP2 RDTs, and 0.987 (95% CI: 0.975–0.999) for the HBsAg RDTs (S1 Fig). The ROC values indicated that the rapid diagnostics had excellent measure of discrimination for the infection.

### Association of age, obstetric and socio-economic determinants with the different groups of study participants

No statistical difference in age was recorded across the different groups (Uninfected, *P*. *falciparum* mono-infection, HBV mono-infection, and *P*. *falciparum*/HBV co-infection) of pregnant women (*p = 0*.*129*; Table 2). Univariate analysis by chi-square test revealed similar proportions of primigravidae and multigravidae across the groups (χ^2^  =  1.98, *p* = 0.576), however, the proportions in gestational age were significantly different (χ^2^  =  39.89, *p*<0.001; Table 2). For the socio-economic parameters considered, involvement in economic activity was independent of the disease categories (χ^2^  =  5.09, *p*<0.165; Table 2). However, the proportions of pregnant with formal education (χ^2^  =  32.8, *p*<0.001) and good financial standings (χ^2^  =  31.35, *p*<0.001) differed significantly across the groups (Table 2).

**Table 2:** Differences in age, obstetric and socio-economic information in study group.

Parameters	Infection type				(*X*^*2*^), ^α^^/^^β^*p*
	Uninfected (*n* = 1602)	*P*. *falciparum* mono-infection (*n* = 278)	HBVmono-infection (*n* = 155)	*P*. *falciparum*/HBV co-infection (*n* = 36)	
**Age, years, mean ± SD**	28.0 ± 5.8	27.4 ± 5.7	27.2 ± 4.8	27.1 ± 5.6	^β^0.129
**Gravidity**					
**Primigravidae, n (%)**	341 (21.3%)	69 (24.8%)	35 (22.6%)	9 (25.6%)	χ^2^ = 1.98, ^α^0.576
**Multigravidae, n (%)**	1261 (78.7%)	209 (75.2%)	120 (77.4%)	27 (77.4%)	
**Gestation**					
**First trimester, n (%)**	421 (76.3%)	51 (9.2%)	68 (12.3%)	12 (2.2%)	χ^2^ = 39.89, ^α^<0.001
**Second trimester, n (%)**	1023 (78.7%)	188 (14.5%)	68 (5.2%)	21 (1.6%)	
**Third trimester, n (%)**	158 (72.1%)	39 (17.8%)	19 (8.7%)	3 (1.4%)	
**Education**					
**Formal, n (%)**	650 (40.7%)	42 (21.4%)	59 (38.1%)	7 (19.4%)	χ^2^ = 32.8, ^α^<0.001
**None, n (%)**	949 (59.3%)	154 (78.6%)	96 (61.9%)	29 (80.6%)	
**Economic activity**					
**Yes, n (%)**	978 (61.2%)	131 (66.8%)	98 (63.2%)	27 (75.0%)	χ^2^ = 5.09, ^α^0.165
**No, n (%)**	621 (38.8%)	65 (33.2%)	57 (36.8%)	9 (25.0%)	
**Financial Status**					
**Good, n (%)**	938 (58.7%)	74 (37.8%)	84 (54.2%)	21 (58.3%)	χ^2^ = 31.35, ^α^<0.001
**Poor, n (%)**	661 (41.3%)	122 (62.2%)	71 (45.8%)	15 (41.7%)	

^α^analyzed using Pearson’s chi-square test and

^β^ analyzed using ANOVA.

χ^2^  = Pearson’s chi-square value

*p* significant at <0.05 (2-tailed)

n = number of pregnant women

Infection type was determined using PCR.

### Factors that predict infection type among study participants

Using multinomial logistic regression analyses, the relative contributions of age, gestation, gravidity and SES as predictors of infection type in the pregnant women were examined. Prior to the analysis, a linear regression model was run to ensure there were no multicolinearity issues among the predictor variables. Only predictors with variance of inflation (VIF) of <2.000 were included in the multinomial regression model (Tables A–G in S1 Table). In addition, correlation among the predictor variables were assessed by Pearson’s test; predictors with Pearson’s correlation coefficient (r) of <0.400 were considered (Table G in S1 Table). Furthermore, the relationship between continuous independent variables (age) and the dependent variable (infection type) was assessed. The results showed that age positively correlated with infection type (r = 0.048, *p = 0*.*03*). Lastly, the data was checked to ensure there were no outliers. The multinomial analyses showed that age did not influence infection type among the pregnant women (Table 3). There was a significant increase in HBV mono-infection among first trimester pregnant women (AOR  =  2.03, 95% CI  =  1.10–3.75, *p* = 0.023, Table 3). Primigravidae had a higher risk for *P*. *falciparum* mono-infection (AOR  =  1.94, 95% CI  =  1.33–2.83, *p* = 0.001, Table 3). SES was observed to influence *P*. *falciparum* mono-infection and *P*. *falciparum*/HBV co-infection, but not HBV mono-infection (Table 3). Particularly, pregnant women who had formal education were at a lower risk for *P*. *falciparum* mono-infection (AOR  =  0.48, 95%CI  =  0.32–0.71, *p*<0.001) and *P*. *falciparum*/HBV co-infection (AOR  =  0.27, 95% CI  =  0.11–0.67, *p* = 0.005; Table 3). Also, those with good financial standing were at a lower risk for *P*. *falciparum* mono-infection (AOR  =  0.52, 95% CI  =  0.36–0.74, *p*<0.001, Table 3).

**Table 3:** Multinomial logistic regression of parameters that predicts infection type in the study participants.

Infection type	Parameters		B	Standard error	Exp(B)/AOR	95% CI AOR	*p*
*P*. *falciparum mono-*infection	Age		-0.007	0.014	0.99	0.97–1.02	0.623
	Gravidity	Primigravidae	0.662	0.193	1.94	1.33–2.83	0.001
		Multigravidae	0^b^				
	Gestation	First trimester	-0.332	0.301	0.72	0.40–1.30	0.271
		Second trimester	-0.158	0.227	0.85	0.55–1.33	0.485
		Third trimester	0^b^				
	Formal education	Yes	-0.741	0.204	0.48	0.32–0.71	<0.001
		No	0^b^				
	Economic activity	Yes	-0.078	0.192	0.93	0.64–1.35	0.685
		No	0^b^				
	financial status	Good	-0.659	0.185	0.52	0.36–0.74	<0.001
		Poor	0^b^				
HBVmono-infection	Age		-0.025	0.016	0.98	0.95–1.01	0.114
	Gravidity	Primigravidae	0.076	0.222	1.08	0.70–1.67	0.732
		Multigravidae	0^b^				
	Gestation	First trimester	0.709	0.313	2.03	1.10–3.75	0.023
		Second trimester	-0.436	0.280	0.65	0.37–1.12	0.119
		Third trimester	0^b^				
	Formal education	Yes	-0.248	0.203	0.78	0.52–1.16	0.222
		No	0^b^				
	Economic activity	Yes	0.068	0.204	1.07	0.72–1.56	0.739
		No	0^b^				
	financial status	Good	-0.411	0.211	0.66	0.44–1.00	0.052
		Poor	0^b^				
*P*. *falciparum*/HBV co-infection	Age		-0.029	0.031	0.97	0.91–1.03	0.347
	Gravidity	Primigravidae	0.640	0.430	1.90	0.82–4.41	0.136
		Multigravidae	0^b^				
	Gestation	First trimester	0.973	0.702	2.65	0.67–10.47	0.166
		Second trimester	0.285	0.63	1.33	0.39–4.59	0.651
		Third trimester	0^b^				
	Formal education	Yes	-1.324	0.48	0.27	0.11–0.67	0.005
		No	0^b^				
	Economic activity	Yes	0.79	0.44	2.21	0.93–5.27	0.379
		No	0^b^				
	Financial status	Good	0.35	0.40	1.42	0.65–3.07	0.136
		Poor	0^b^				

*p*: analyzed by multinomial logistic regression analyses, and considered significant at < 0.05 (2-tailed).

The reference category is: Uninfected.

b: parameter considered redundant and set to zero

B: regression coefficient.

ExpB: exponentiation of B, which is the same as AOR: Adjusted odds ratio.

95% CI: 95% confidence interval

## Discussion

In Ghana high prevalence rates of malaria and hepatitis B have been reported among pregnant women attending ANC. In many endemic settings including the study sites, these infections are diagnosed by the use of HRP2 and HBsAg RDTs as these allow quick determination of results. However, to ascertain the reliability of these RDTs in reporting the prevalence of malaria and hepatitis B, it is paramount to evaluate the performance of these diagnostics with a more sensitive and specific diagnostic tool such as PCR. To address this issue, a population of pregnant women in the Tamale Metropolis and Central Gonja District of the Northern Region of Ghana were studied because of their high ANC performance compared to other areas in the country [24].

Though no significant statistical differences in prevalence rates were observed between the RDTs and the PCRs, the marginal differences recorded by the RDTs could be ascribed to false positivity. In the case of malaria, reasons that could be advanced for this observation would include delayed clearance of HRP2 after parasite clearance [10, 11]or cross reactivity with other infections such as typhoid, schistosomiasis and hepatitis C virus [12–14]. In the case of HBV infection evidence abounds of false positive HBsAg reactivity; which is well known and attributed to recent hepatitis B vaccination [15, 16]. That notwithstanding, the RDTs performed excellently in discriminating for the infections when compared with the PCRs. This suggests that the RDTs are reliable and can give the representative picture of the prevalence of malaria and hepatitis B in endemic countries. However, the marginal differences shown between RDT and PCR prevalence rates could translate into large numbers of pregnant women mis-diagnosed over a long period and for that matter mistreated. Therefore, results obtained from these RDTs should be confirmed by other diagnostics such microscopy for malaria parasites, serological and laboratory-based EIAs for HBV or PCR [27], so as to inform treatment options.

The study reports a PCR prevalence of 15.2% (Table 1) for malaria, which is comparable with the national figure of 17.6% [2]. With the current effective vector management strategies such free distribution of insecticide treated bed nets; that protects from mosquito bites, it is expected that the prevalence of malaria in pregnancy should significantly decline. However, recent evidence indicating that malaria in pregnant women on first antenatal visits does not require recent exposure to infected mosquitoes throws light that insecticide treated bed nets may not protect against malaria in pregnancy [28].The PCR prevalence of 9.2% for hepatitis B amongst the pregnant women (Table 1), is >8% and is therefore suggestive of high carriage of a chronic HBV with a high risk of liver disease progression [29, 30]. Thus, the disease remains a significant public health issue worth addressing. The study reports an increased prevalence (1.7% by PCR) of *P*. *falciparum*/HBV co-infection compared to the rate of 0.7% reported among pregnant women in northern Ghana [31]. This sends a wake-up call to health institutions and authorities of endemic regions, because it is not known whether this co-infection could have profound effects on pregnancy and pregnancy outcomes. However, based on reports that malaria, viral infections and anemia are indirect causes of pregnancy-related deaths [32], co-infection with *P*. *falciparum* and HBV could aggravate adverse obstetric outcomes.

Starting ANC in the first trimester and coupled with the WHO updated model which is based on reduced but goal-orientated clinic visits [33], is paramount in preventing pregnancy related complications and maternal mortalities [34]. This most especially, is essential for primigravidae, because they have an increased risk for placental malaria [18]; which was also evidenced in this study. Reasons abound to the fact that they are not previously exposed and therefore lack specific immunity to placental malaria [35, 36].It was observed that first trimester pregnant women had a higher risk for HBV mono-infection, which may be due to the uneven distribution in gestational ages across the different disease groups. Despite the benefits of early ANC, the study found large proportions of the pregnant women turning out for ANC in the second trimester, which is common among African women and can be explained by various reasons [37]. There is therefore the need to strengthen education on all fronts to encourage early and routine ANC visits by pregnant women.

Evidence from our study revealed that high SES (formal education and/or good financial standing) was associated with lower risk of *P*. *falciparum* mono-infection and *P*. *falciparum*/HBV co-infection among the pregnant women. This finding buttresses the point that improving SES of people in endemic areas would enormously help in reducing incidence of malaria [23]. However, the study found no influence of SES on the prevalence of HBV mono-infection, which may be explained by the fact that knowledge and awareness of hepatitis B is generally low in the Ghanaian population, irrespective of social class [38–41]. Therefore, raising awareness of hepatitis B in all socio-economic facets of the Ghanaian population, could help reduce the burden of the disease and co-infection with malaria. The knowledge of hepatitis B vaccination status of pregnant women at ANC would be helpful in reducing incidence of HBsAg false positivity. In addition, the initiation of routine screening of pregnant women for both hepatitis B and malaria at each ANC visit, would contribute greatly to the timely management of cases and prevention of any possible adverse effects that might be associated with *P*. *falciparum*/HBV co-infection.

## Limitations

The study was not without some limitations. Firstly, the performance of only one brand of HRP2 and HBsAg RDTs was evaluated for reporting the prevalence of the infections, thus, the findings may not be reflective of other commercially available RDTs. Also, only serum samples were tested for the presence of HBsAg, prompting the need to assess the performance of the HBsAg RDTs from whole blood samples. In addition, the study may not account for the impact of HIV-seropositivity on performance of the HBsAg RDTs, however, it is reported that specificity of HBsAg RDTs remained high in studies that compared performance between HIV-positive and HIV-negative persons [8].

## Conclusions

Our data has shown that, the RDTs are comparable to PCR and can give a representative picture of the prevalence of malaria and hepatitis B in endemic countries. Also, our results support the facts that improving SES is paramount in eliminating malaria in endemic settings. However, SES did not influence the prevalence of HBV mono-infection among pregnant women in northern Ghana.

## Supporting information

S1 File Seroprevalence of Malaria and Hepatitis B Coinfection amongPregnant Women in Tamale Metropolis of Ghana. A Cross-Sectional Study (Helegbe et al., 2018).
(PDF)

S1 Fig **Receiver operator curve of**: (A) HRP2 RDTs compared with PCR for the detection of *P*. *falciparum*, and (B) HBsAg RDTs compared with PCR for the detection of HBV.
(TIF)

S1 Table Collinearity analyses with socio-demographic variables and correlations between predictor variables. (DOCX)
