# Supplementary figures and images for: Prevalence of malaria and hepatitis B among pregnant women in Northern Ghana: Comparing RDTs with PCR
Source: PLoS One. 2019 Feb 6;14(2):e0210365. doi: 10.1371/journal.pone.0210365 (PMC6364880; doi:10.1371/journal.pone.0210365)

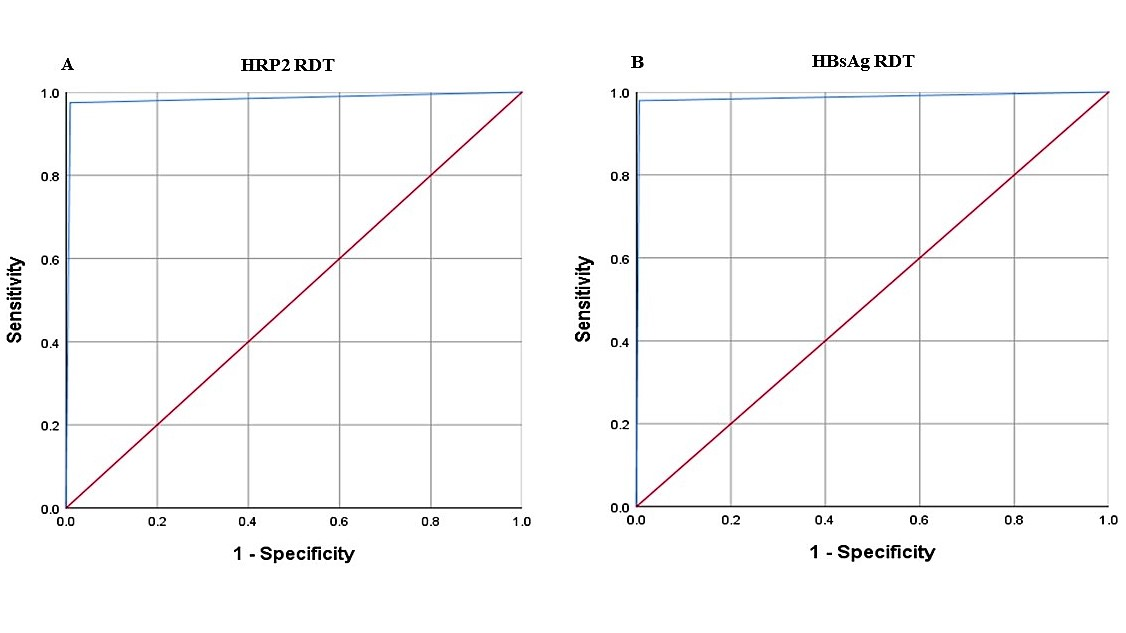

Supplement: S1 Fig — Receiver operator curve of: (A) HRP2 RDTs compared with PCR for the detection of P. falciparum, and (B) HBsAg RDTs compared with PCR for the detection of HBV. (TIF) [file pone.0210365.s002.TIF]
