# Supplementary material for: Prevalence of malaria and hepatitis B among pregnant women in Northern Ghana: Comparing RDTs with PCR
Source: PLoS One. 2019 Feb 6;14(2):e0210365. doi: 10.1371/journal.pone.0210365 (PMC6364880; doi:10.1371/journal.pone.0210365)
Supplement: S1 Table — (DOCX) [file pone.0210365.s003.docx]

**SUPPLEMENTARY TABLES**

Table A: Collinearity analyses with age as dependent variable

| Predictor | B | Standard error | Beta | Collinearity statistics | |
| --- | --- | --- | --- | --- | --- |
|  |  |  |  | Tolerance | VIF |
| Gravidity | -0.193 | 0.330 | -0.014 | 0.856 | 1.168 |
| Gestation | 0.381 | 0.241 | 0.040 | 0.793 | 1.260 |
| Educational status | -0.175 | 0.297 | -0.015 | 0.769 | 1.301 |
| Economic status | -0.283 | 0.301 | -0.024 | 0.750 | 1.334 |
| Financial status | -1.751 | 0.298 | -0.153 | 0.730 | 1.370 |

B: unstandardized regression coefficient

Beta: standardized regression coefficient

VIF: variance inflation factor

Table B: Collinearity analyses with gravidity as dependent variable

| Predictor | B | Standard error | Beta | Collinearity statistics | |
| --- | --- | --- | --- | --- | --- |
|  |  |  |  | Tolerance | VIF |
| Gravidity | 0.041 | 0.016 | 0.059 | 0.795 | 1.258 |
| Gestation | 0.061 | 0.020 | 0.071 | 0.772 | 1.295 |
| Educational status | -0.268 | 0.020 | -0.314 | 0.820 | 1.220 |
| Economic status | 0.021 | 0.020 | 0.025 | 0.718 | 1.393 |
| Financial status | -0.001 | 0.002 | -0.012 | 0.980 | 1.021 |

B: unstandardized regression coefficient

Beta: standardized regression coefficient

VIF: variance inflation factor

Table C: Collinearity analyses with gestation as dependent variable

| Predictor | B | Standard error | Beta | Collinearity statistics | |
| --- | --- | --- | --- | --- | --- |
|  |  |  |  | Tolerance | VIF |
| Gravidity | 0.247 | 0.027 | 0.204 | 0.801 | 1.249 |
| Gestation | -0.090 | 0.028 | -0.074 | 0.753 | 1.328 |
| Educational status | 0.330 | 0.027 | 0.278 | 0.772 | 1.296 |
| Economic status | 0.003 | 0.002 | 0.032 | 0.981 | 1.020 |
| Financial status | 0.077 | 0.031 | 0.054 | 0.859 | 1.164 |

B: unstandardized regression coefficient

Beta: standardized regression coefficient

VIF: variance inflation factor

Table D: Collinearity analyses with educational status as dependent variable

| Predictor | B | Standard error | Beta | Collinearity statistics | |
| --- | --- | --- | --- | --- | --- |
|  |  |  |  | Tolerance | VIF |
| Gravidity | -0.167 | 0.022 | -0.167 | 0.770 | 1.298 |
| Gestation | 0.236 | 0.022 | 0.241 | 0.759 | 1.318 |
| Educational status | -0.001 | 0.002 | -0.012 | 0.980 | 1.021 |
| Economic status | 0.075 | 0.025 | 0.064 | 0.860 | 1.163 |
| Financial status | 0.163 | 0.018 | 0.198 | 0.826 | 1.211 |

B: unstandardized regression coefficient

Beta: standardized regression coefficient

VIF: variance inflation factor

Table E: Collinearity analyses with economic status as dependent variable

| Predictor | B | Standard error | Beta | Collinearity statistics | |
| --- | --- | --- | --- | --- | --- |
|  |  |  |  | Tolerance | VIF |
| Gravidity | -0.216 | 0.022 | -0.221 | 0.753 | 1.329 |
| Gestation | -0.002 | 0.002 | -0.019 | 0.980 | 1.021 |
| Educational status | -0.322 | 0.024 | -0.275 | 0.937 | 1.067 |
| Economic status | -0.058 | 0.018 | -0.070 | 0.797 | 1.255 |
| Financial status | -0.162 | 0.022 | -0.163 | 0.790 | 1.266 |

B: unstandardized regression coefficient

Beta: standardized regression coefficient

VIF: variance inflation factor

Table F: Collinearity analyses with financial status as dependent variable

| Predictor | B | Standard error | Beta | Collinearity statistics | |
| --- | --- | --- | --- | --- | --- |
|  |  |  |  | Tolerance | VIF |
| Gravidity | 0.025 | 0.025 | 0.021 | 0.996 | 1.004 |
| Gestation | 0.212 | 0.017 | 0.252 | 0.857 | 1.167 |
| Educational status | 0.229 | 0.022 | 0.225 | 0.852 | 1.174 |
| Economic status | -0.216 | 0.022 | -0.211 | 0.812 | 1.231 |
| Financial status | 0.025 | 0.025 | 0.021 | 0.786 | 1.272 |

B: unstandardized regression coefficient

Beta: standardized regression coefficient

VIF: variance inflation factor

Table G: Correlations between predictor variables

| predictor variables | | Age | Gravidity | Gestation | Educational status | Economic status | Financial status |
| --- | --- | --- | --- | --- | --- | --- | --- |
| Age | r | 1 | -0.030 | -0.016 | -0.056 | 0.031 | -0.137 |
|  | *p* | - | 0.169 | 0.471 | 0.012 | 0.169 | <0.001 |
|  | n | 2071 | 2071 | 2071 | 1986 | 1986 | 1986 |
| Gravidity | r | -0.030 | 1 | 0.180 | 0.206 | -0.362 | 0.192 |
|  | *p* | 0.169 | - | <0.001 | <0.001 | <0.001 | <0.001 |
|  | n | 2071 | 2071 | 2071 | 1986 | 1986 | 1986 |
| Gestation | r | -0.016 | 0.180 | 1 | 0.347 | -0.261 | 0.391 |
|  | *p* | 0.471 | <0.001 | - | <0.001 | <0.001 | <0.001 |
|  | n | 2071 | 2071 | 2071 | 1986 | 1986 | 1986 |
| Educational status | r | -0.056 | 0.206 | 0.347 | 1 | -0.329 | 0.392 |
|  | *p* | 0.012 | <0.001 | <0.001 | - | <0.001 | <0.001 |
|  | n | 1986 | 1986 | 1986 | 1986 | 1986 | 1986 |
| Economic status | r | 0.031 | -0.362 | -0.261 | -0.329 | 1 | -0.362 |
|  | *p* | 0.169 | <0.001 | <0.001 | <0.001 | - | <0.001 |
|  | n | 1986 | 1986 | 1986 | 1986 | 1986 | 1986 |
| Financial status | r | -0.137 | 0.192 | 0.391 | 0.392 | -0.362 | 1 |
|  | *p* | <0.001 | <0.001 | <0.001 | <0.001 | <0.001 | - |
|  | n | 1986 | 1986 | 1986 | 1986 | 1986 | 1986 |

*p:* analyzed by Pearson’s correlation test, and considered significant at <0.05 (2-tailed)

r: Pearson’s correlation coefficient

n: number of participants

-: not computed
